# Effect of Lentinan on Lipid Oxidation and Quality Change in Goose Meatballs during Cold Storage

**DOI:** 10.3390/foods11071055

**Published:** 2022-04-06

**Authors:** Li Fu, Lihui Du, Yangying Sun, Xiankang Fan, Changyu Zhou, Jun He, Daodong Pan

**Affiliations:** 1 State Key Laboratory for Managing Biotic and Chemical Threats to the Quality and Safety of Agro-Products, Ningbo University, Ningbo 315211, China; fufufuli1997@163.com (L.F.); dulihui@nbu.edu.cn (L.D.); sunyangying@nbu.edu.cn (Y.S.); fanxiankang2022@163.com (X.F.); zhouchangyu@nbu.edu.cn (C.Z.); hejun@nbu.edu.cn (J.H.); 2 Key Laboratory of Animal Protein Food Processing Technology of Zhejiang Province, College of Food and Pharmaceutical Science, Ningbo University, Ningbo 315832, China

**Keywords:** fungus polysaccharide, antioxidant, goose meat product, lipid oxidation, flavor substances

## Abstract

The effects of different concentrations of lentinan (LNT) (0, 0.5, 1, 2 and 4%) on the oxidation characteristics and physicochemical properties of goose meatballs were investigated during different cold storage (4 °C) stages (3, 7 and 12 days). After adding LNT, the thiobarbituric acid reactive substances (TBARS) and total volatile base nitrogen (TVB-N) of goose meatballs significantly decreased compared to the LNT-free sample during cold storage, which indicated that LNT can inhibit the fat oxidation and the release of nitrogenous substances. Meanwhile, the presence of LNT makes microstructure of the goose meatball samples become denser during the whole storage time. The headspace solid phase microextraction gas chromatography-mass spectrometry (SPME-GC-MC) results showed that the proportion of aldehydes in the 4% LNT group reached 0 during storage, suggesting that high LNT concentration inhibits the formation of oxidized products in meat products. The sensory evaluation showed that the addition of LNT improved the color, appearance, flavor, and overall acceptance of goose meatballs, and the 2% LNT group had the highest score in overall acceptance. In summary, the addition of LNT could delay lipid oxidation and improve the quality of goose meatballs during cold storage.

## 1. Introduction

China has the largest volume of goose meat production; about 2.52 million tons of goose meat is consumed annually [1]. From a nutritional point of view, goose meat is rich in protein; its protein content is as high as 22.3%. It contains all the essential amino acids that needed for human body [2]. The fat content of goose meat is 3–4% and it is mainly composed by unsaturated fatty acid, in which the linolenic acid shows higher content when compared with other meat varieties [3,4]. Goose meat also contains low cholesterol (52–76 mg/100 g) and a variety of vitamins, niacin, sugar, and minerals [4]. Except for the superior nutritional value, goose meat has certain advantages in appearance, texture, and taste in comparison. Gulbaz and Kamber [5] found that the panelists gave raw goose sausage higher sensory scores for appearance, flavor, texture, and color than raw beef sausage. Hamadani et al. [6] found that goose meat presented more market space in terms of overall acceptance among goose, chicken, and mutton. Therefore, various goose meat products enter the market in order to meet people’s demand for high quality poultry products, such as goose meatballs, goose sausage, dry pot goose feet etc. However, the unsaturated fatty acids, which account for 70% of the total fatty acids in goose meat [4], can be easily oxidized during processing and refrigeration, resulting in peculiar smell, deterioration and reduction in quality [7]. Wereńska et al. [8] investigated the oxidation activity and lipid oxidation changes in breast and leg muscles of goose, severe oxidation occurred after 365 days of frozen storage and the intense gray-brown color of meat was not accepted by the consumer. Therefore, measures need to be taken to alleviate the oxidation process of the goose meat products.

At present, a large number of chemical synthetic antioxidants have been added to meat products as exogenous additives in order to inhibit the fat oxidation, for instance, Butylated hydroxy anisole (BHA), Butylated hydroxytoluene (BHT) and Propyl Gallate (PG), which may bring negative effects to consumers’ health [9]. As a result, seeking natural antioxidants that can extend the shelf-life of meat products is an important topic. Some natural antioxidants that are rich in sugars and phenols have been reported to show decent antioxidant activity. The natural extracts from green tea [10], pitanga leaf [11], chrysanthemum [12] have been used in meat products to reduce the oxidation therefore preserve the meat quality. At the same time, since poultry products usually have an unpleasant fishy smell, adding antioxidants from natural sources can effectively enhance the flavor characteristics [13]. Hayes et al. [14] found that adding grape seed extract to chicken products during manufacturing can effectively improve the sensory properties of chicken and delay the lipid oxidation process. Hozová et al. [15] found that by adding LNT to the yogurt, the samples maintained a very good quality throughout the storage, thus representing an effective alternative to synthetic antioxidants to be used during food processing and storage.

Lentinus edodes is a fungus that has long been consumed as food. It has been proved that Lentinus edodes had high nutritional value and showed great pharmacological activity [16]. Lentinan (LNT), which is mainly extracted and purified from Lentinus edodes, is an important active ingredient of Lentinus edodes. LNT has many functions such as anti-oxidation, anti-tumor, anti-inflammation, immune regulation, etc. [17]. Olawuyi and Lee [18] reported that the addition of Lentinus edodes powder to the rice muffins enhanced the antioxidant activity and improved the flavor of the rice muffins. However, the application of LNT in meat products is rarely reported and there is no study concerning the LNT effect in goose meat products to date.

Therefore, the purpose of this study was to understand the influence of LNT content and storage time on the quality of goose meatballs at 4 °C by measuring and analyzing pH value, color, thiobarbituric acid active substances (TBARS), total volatile base nitrogen (TVB-N), texture, microstructure, flavor, and sensory characteristics of the samples.

## 2. Materials and Methods

### 2.1. Materials and Samples

Fresh goose (the eviscerated carcass weighs about 3 kg) was purchased from Beilun Food Co., Ltd. (Ningbo, China), and removed goose breast meat as raw material for goose meatballs. Lentinan (50%) was purchased from Zhongzhikang mushroom biotechnology Co., Ltd. (Hangzhou, China). All seasonings were obtained from local supermarkets (Ningbo, China). All chemicals and reagents were purchased from Macklin Biochemical Co., Ltd. (Shanghai, China). All chemicals used were of an analytical grade.

### 2.2. Preparation of Goose Meatballs

The visible connective tissues were firstly removed from the goose breast meat and the meat was cut into small pieces before placed in a meat grinder for 120 s, meanwhile, different concentrations of LNT (0, 0.5, 1, 2, and 4%) were mixed with pig backfat (20.0%), salt (2.0%), sugar (1.0%), light soy sauce (3.0%), edible oil (3.0%), cooking wine (2.0%), thirteen spices (1.0%), high gluten powder (10.0%), hot water (10.0%), and monosodium glutamate (1.0%) (the percentage above refers to the proportion of raw meat) to form a uniform paste. Finally, the seasoned meat was shaped into goose meatballs with a weight of 20.0 g, followed by setting in cold water bath (25 °C) for 30 min and boiling (100 °C) for 20 min. After cooling, the goose meatballs were packed in a vacuum bag and placed the refrigerator (4 °C) before analysis.

### 2.3. pH and Color 

The pH was measured by a pH meter (FE20, Mettler Tolrdo, Shanghai, China) according to the method of Jin & Choi [19]. A total of 5 g sample was homogenized (60 s, 10,000 rpm) (XHF-D, Xinzhi Biotechnology Co., Ltd., Ningbo, China) in 20 mL of distilled water. The pH meter was calibrated with standard buffers of pH 4.0, 7.0 and 10.0 at 25 °C before using.

The color of sample was measured using a colorimeter (SWG-2300, Shuo Guang Electronic Technology Co., Ltd., Shanghai, China) with illuminant D65 at the observation angle of 10°, following the method of Chai and Sheen [20]. The CIEL*a*b* values (measures lightness, redness, and yellowness, respectively) of each sample were directly recorded from the exposed surface after the goose meatballs were cut into 15 mm slice. The instrument was standardized using a white plate before the measurements. The total color change (ΔE) was calculated using the following formula:

(1) $$\Delta E=[{\left(L^{*}\;-\;L_{0}^{*}\right)}^{2}+{\left(a^{*}\;-\;a_{0}^{*}\right)}^{2}+{\left(b^{*}\;-\;b_{0}^{*}\right)}^{2}{]}^{1/2}{=[(\Delta L)}^{2}{+(\Delta a)}^{2}{+(\Delta b)}^{2}{]}^{1/2}$$

where L*, a*, and b* were obtained from the tested samples and L_0_*, a_0_*, and b_0_* were measured from the control samples.

### 2.4. Thiobarbituric Acid Reactive Substances (TBARS)

TBARS were estimated according to the current Chinese standard method for food (GB/T 5009.181-2016); five grams of the ample was homogenized (60 s, 10,000 rpm) in trichloroacetic acid (50 mL, 10% (*w/v*) TCA), and shaken at 50 °C for 30 min by the shaker (HZ-2210K-2, Taicang Hualida Experimental Equipment Co. Ltd., Jiangsu, China). The obtained sample was filtrated with Jiaojie NO.101, 10 milliliters of filtrate were taken, mixed with thiobarbituric acid (TBA, 10 mL, 0.02 mol/L) and placed in a Dk-8d three-hole electric thermostatic water bath (90 °C) (Yiheng Scientific Instrument Co., Ltd., Shanghai, China) for 40 min. The mixture was then cooled to room temperature (25 °C) and the absorbance was measured at 532 nm using a Spectrophotometer (P4, Meipuda Instrument Co., Ltd., Shanghai, China) against a blank containing 10 mL of TCA and 10 mL of TBA solution. Results were expressed as mg of malondialdehyde (MDA) per kilogram of goose meatballs sample.

(2) $$\mathrm{TBARS}(\mathrm{mg}/\mathrm{kg})=\frac{\;c\;\times \;V}{M}$$

c represents the concentration of malondialdehyde in the sample solution obtained from the standard series curve (μg/mL). V represents the constant volume of the sample solution. M represents the sample mass in the final sample solution.

### 2.5. Total Volatile Base Nitrogen (TVB-N)

TVB-N content was measured according to the current Chinese standard method for food (GB/T 5009.228-2016). Ten grams of the sample was homogenized (36 s, 10,000 rpm) with 100 mL of cold distilled water. After soaking for 30 min, the sample was filtrated by Jiaojie NO.101 filter paper. The 10 mL filtrate was distilled after the addition of 5 mL MgO (10 g/L) using an automatic Kjeldahl Apparatus (Hanon Instruments Co., Ltd., Jinan, China). The distillate was collected in a flask containing 10 mL of 20 g/L boric acid with methyl red-bromocresol green mixed indicator. Then, the boric acid solution was titrated with a 0.01 mol/L sulfuric acid solution, and the TVB-N was expressed as mg nitrogen/100 g muscle.

(3) $$\mathrm{TVB-N}\;\left(\mathrm{mg}/100\;g\right)=\frac{\left(V_{1}\;{-\;V}_{2}\right)\;\times \;c\;\times \;14}{m\;\times \;\left({V/V}_{0}\right)}\;\times \;100$$

V_1_ represents the volume of hydrochloric acid or sulfuric acid standard titration solution consumed by the test solution, mL. V_2_ represents the volume of standard titrated solution of hydrochloric acid consumed by reagent blank, mL. c represents the concentration of standard titrated solution of hydrochloric acid, 0.01 mol/L. The number 14 represents the mass of nitrogen equivalent to a standard titrated solution of 1.0 mL hydrochloric acid, in grams per mole (g/mol). M represents the sample mass. V represents the volume of filtrate accurately absorbed. V_0_ represents the total volume of sample solution.

### 2.6. Scanning Electron Microscope (SEM)

The microstructure of samples was inspected by a SEM (S3400, Hitachi, Japan) according to the method of Zhou et al. [21] with slight modification. The sample was cut into pieces (5 mm × 3 mm × 3 mm), and then frozen at liquid nitrogen for 24 h, followed by lyophilization using a freeze dryer (Alpha 1–4 LD plus, CHRIST, Germany). The lyophilized samples were vacuum ion sputtered with a gold plating film (150 s) and the microstructure of the sample was observed at 200× magnifications at an accelerating voltage of 10.0 kV. Each treatment was analyzed in three replicates.

The microstructure pictures were edited by ImageJ v1.51 to calculate the fractal dimension (Df) and to obtain the binarization image of the samples. Images were processed to binary images by adjusting the threshold [22]. The Fractal dimension of the image is obtained by using the Fractal Box Count plug-in. Three replicates were performed for each sample.

### 2.7. Texture Profile Analysis (TPA)

TPA was carried out using a texture analyzer (XT Plus, Stable Micro Systems Ltd., God alming, UK). The testing parameters were referred to Reihami et al. [23] with slight modification. The calibration was performed using a 5 kg load cell and distance before analysis. TPA was performed with a cylindrical probe (diameter 50 mm). The meatball sample was cut into a cylinder with a height of 15 ± 0.5 mm and placed on the middle of the compression plate and was determined with the TPA mode. Two continuous compressions were carried out. The instrument setting was: Trigger type,10 g; Pre-test speed, 2.0 mm/s; Test speed, 1 mm/s; Post-test speed, 1 mm/s; Strain, 50%; Interval between two compression, 5 s. The measured results are expressed by hardness, springiness, cohesiveness, and chewiness. 

### 2.8. Electronic Tongue Analysis

The analysis was performed using an electronic tongue system (SMARTONGUE Ruifin Intelligent Technology Co., Ltd., Shanghai, China). Some modifications were made based on Dang et al. [24]. The samples were weighed (6.00 g) and homogenized (12 s, 10,000 rpm) with 75 mL of deionized water and was allowed to stand at 37 °C for 30 min. Filter with Jiaojie NO.101: fifteen millimeters of filtrate was poured into the sampling cup before analysis. Six replicates were performed for each group of samples, and three were selected for principal component analysis (PCA). PCA was performed according to the instrument software. 

### 2.9. Analysis of Volatile Compounds

The volatile compounds in the goose meatballs were extracted and analyzed using a headspace solid phase microextraction gas chromatography-mass spectrometry (SPME-GC-MS,8890 GC System+5977B/MSD, Agilent Technologies Inc., NewYork, NY, USA) as described by Deng et al. (2021) [25] with some modifications. Moreover, 5 grams of sample was shredded and transferred into a 20 mL extraction bottle while the internal standard 2-methyl-3-heptanone (20 ppm, 5 μL) was added. The 50/30 μm divinylbenzene/carboxen/polydimethylsiloxane (DVB/CAR/PDMS) extraction head was inserted into a sealed extraction bottle and extracted at 60 °C for 40 min. The extraction head was thermally analyzed at 250 °C at the gas phase injection port for 5 min. The flavor components were then analyzed by a gas chromatography-mass spectrometry. Chromatographic strip: HP-5MS elastic quartz capillary column (30 m × 0.25 mm, 0.25 μm), carrier gas: helium, flow rate 1.0 mL/min, injection without shunt, injection port temperature 250 °C, heating program: initial temperature 40 °C, 5 min; 5 °C/min to 180 °C, then 15 °C/min to 250 °C, 8 min. Mass spectrometry conditions: EI ion source, ion source temperature 230 °C, electron energy 70 eV; mass scanning range 35~500 amu.

The software QualitativeAnalysisB.07.00 was used to match the GC-MS spectrum of volatile substances and the retrieval spectrum database of NIST14.0 Library compounds one by one, and the automatic integral chromatographic peak extraction mass spectrometry was established, and the results with a score of 60 was selected The volatile flavor compounds were quantitatively analyzed according to the known internal standard 2-methyl-3-heptanone content, and according to the principle that the peak area ratio of the compound was proportional to the content, the content of each flavor compound relative to the internal standard compound was calculated.

### 2.10. Sensory Evaluation 

A sensory evaluation of meatballs was implemented according to Ramle et al. [26] by a group of 10 members (acceptability test). The sensory evaluation was scored using a nine-point hedonic scale (1 = “dislike extremely”, 2 = “dislike very much”, 3 = “dislike moderately”, 4 = “dislike slightly”, 5 = “neither like nor dislike”, 6 = “like slightly”, 7 = “like moderately”, 8 = “like very much”, and 9 = “like extremely”). The panelists were randomly selected from students and faculty members with expertise in the research group, and the sensory characteristics of goose meatballs were performed in one session. The meatballs were placed at about 25 °C, cut into 1 cm thickness sections and distributed on white plates before being presented to the group. Each treatment is identified by a three-digit random code. The sensory attributes assessed were color, appearance, elasticity, flavor, taste and over acceptance of the meatball samples. 

### 2.11. Statistical Analysis

The entire experiment was replicated 3 times and each analysis was conducted in triplicate. The results of the pH, color, TBRAS, TVB-N, Df and TPA parameters (hardness, springiness, cohesiveness, and chewiness) were analyzed using two-way analysis of variance (ANOVA), and the contents of volatile flavor substances were analyzed by one-way ANOVA. The comparison among means was evaluated by performing the Duncan’s multiple range tests at a significance level of 0.05. All data were analyzed using the Statistical Package for Social Sciences, Version 26 for Windows (SPSS Inc., Chicago, IL, USA) and data was plotted using OriginPro 2021. The Pearson’s correlation coefficient between dependent variables of goose meatballs was analyzed using the correlation plot of the plug-in in OriginPro 2021.

## 3. Results and Discussion

### 3.1. pH and Color 

pH value is an important indicator to measure meat quality because it directly affects protein stability and protein functionalities [27]. The LNT content (*p* < 0.001), storage time (*p* < 0.001) and LNT content × storage time (*p* < 0.001) significantly influenced the pH in the tested goose meatball (Table S1). As Table 1 shows, the pH value increased first (from day 3 to day 7) and then decreased (from day 7 to day 12) during the storage time. The initial increase in pH may be due to the action of microorganisms and endogenous enzymes that promote the decomposition of meat protein to produce alkaline substances [28]. However, microbial metabolism, such as that of lactic acid bacteria, produces more acid, resulting in a decrease in pH of goose meatballs afterwards [19]. On the other hand, with the increase in LNT content during cold storage, the pH of goose meatballs generally showed a decreasing trend. The pH was significantly reduced even at 0.5% level of LNT stored for 3 days compared to the sample without LNT, and the pH of 4% LNT group dropped the most from 6.073 to 5.930, which may be owing to the acidic nature of LNT. In addition, it is also possible that the presence of LNT inhibited the growth of microorganisms, thus reduced the production of alkaline substances, resulting in the decrease in pH value. However, there was no significant difference among the pH of LNT samples stored for 12 days where the pH tended to be stabilized.

Color is an important indicator to judge food quality [29]. The effects of LNT content and storage time on color changes in goose meatballs during cold storage (4 °C) are shown in Table 1. For meat products, the most important and intuitive parameter in color is the L* and a* values, because it is often associated with consumers’ acceptance of meat color [30]. The L* value of the samples stored for 12 days significantly decreased compared to the samples with 3 and 7 days of storage. And, with the extension of storage days (from 3 days to 12 days), the a* value of goose meatballs generally showed a decreasing trend, however, when the addition of LNT was 0.5%, 1% and 2%, the a* of the samples did not show significant difference. It is understood that proteins and lipids undergo oxidation and degradation during storage, resulting in structural changes that alter the absorption and scattering of light in goose meatballs [31]. In addition, oxidation products such as hydrogen peroxide, ketones, acids, and aldehydes also affect color [32]. With the increase in LNT concentration, L* value showed a downward trend during the whole storage period, which may be the fact that LNT is a brown powder that dispersed in the goose meatballs during production process, therefore excessive addition of LNT caused a decrease in the L* value of sample. However, the a* value showed an increasing trend with the increase in LNT content during storage, specifically, compared with the samples without LNT, the a* of the 4%LNT group increased by 28.15%, 48.50% and 100.03%, respectively, during storage for 3, 7 and 12 days, which indicated that LNT could inhibit the oxidation of fat. LNT has antioxidant activity and can effectively prevent myoglobin from oxidizing to high-valent iron myoglobin [33]. In addition, the increase in LNT concentration had little effect on the b* value of the samples compared to the samples without LNT addition. However, there was significant interaction effect of the LNT content × storage time for b* of the tested sample (*p* < 0.001) (Table S1). Total color difference (ΔE) is a common method to show the magnitude of position difference in CIE LAB color systems and the higher ΔE, the greater the relative change in color and the paler the color than the control [20]. With the increase in LNT content, ΔE showed a rising trend during the whole cold storage period, indicating that adding LNT could change the total color of goose meatballs. Specifically, the higher LNT content (2% LNT and 4% LNT), the more significant the color change in sample goose meatballs. Interestingly, sensory results showed that the presence of LNT increased people’s ratings of the color attributes of goose meatballs throughout storage compared to samples without LNT (Figure S1). 

### 3.2. TBARS

**Figure 1:**
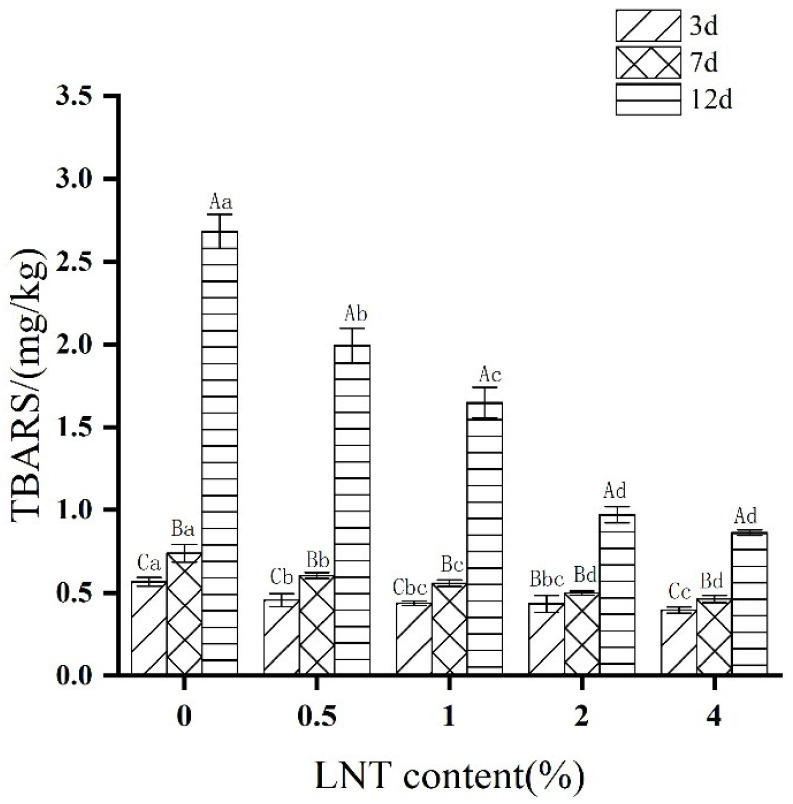
Effects of LNT content and storage time on TBARS of goose meatballs during cold storage (4 °C). Capital letters indicate significant differences between different storage times for the same sample (*p* ≤ 0.05). Lowercase letters indicate significant differences between different contents of LNT sample during the same storage time (*p* ≤ 0.05).

TBARS is often used to evaluate the degree of lipid oxidation and rancidity of meat products during cold and frozen storage [34]. The LNT content (*p* < 0.001), storage time (*p* < 0.001) and LNT content × storage time (*p* < 0.001) significantly influenced the TBARS in the tested goose meatball (Table S1). The effects of LNT content and storage time on TBARS of goose meatballs during cold storage (4 °C) are shown in Figure 1. In general, the TBARS of each sample showed an upward trend with the increase in the storage time, and the TBARS of each treatment group decreased gradually with the increase in the amount of LNT in goose meatballs. The TBARS of goose meatballs with LNT addition was always lower than that of the LNT-free group no matter at which storage time. The TBARS of the LNT sample group decreased by 19–30% compared to the LNT-free group and reached to the maximum reduction (30%) at the 4% LNT group stored for 3 days, indicating that the addition of LNT could inhibit the fat oxidation of goose meatballs. In the study regarding the effect of polysaccharides from *Porphyra yezoensis* (8 g/L) on Pacific white shrimp during storage, a 28.7% reduction in TBA was observed [35]. There was no significant difference in TBARS content between 2% LNT group and 4% LNT group stored for 7 and 12 days. This result indicates that the addition of excessive LNT does not have a dose effect on the oxidation of the sample during the later stage of storage. Feng et al. [36] reported that when the TBA exceeded 1 mg/kg, the sensory properties of the sample were unacceptable and the sample showed a rotten smell. The TBARS of control group, 0.5% LNT group, 1% LNT group, 2% LNT group and 4% LNT group were 2.66, 1.99, 1.65, 0.97, and 0.86 mg/kg during the 12 days of storage, respectively. It illustrates that in the later stage of storage, the addition of LNT can effectively inhibit the fat oxidation of goose meatballs and help to prevent the formation of off-flavor. This may because LNT is able to slow down the chain reactions initiated by free radicals [37], and inhibit catalytic oxidation as well as the reactions with lipid peroxides [38]. Meanwhile, Xue & He [39] found that adding 3% chicory polysaccharide significantly decreased the oxidation of lipids in silver carp. Hamzaoui et al. [40] reported that the addition of green algae polysaccharide in beef sausage lowered the content of TBARS.

### 3.3. TVB-N

**Figure 2:**
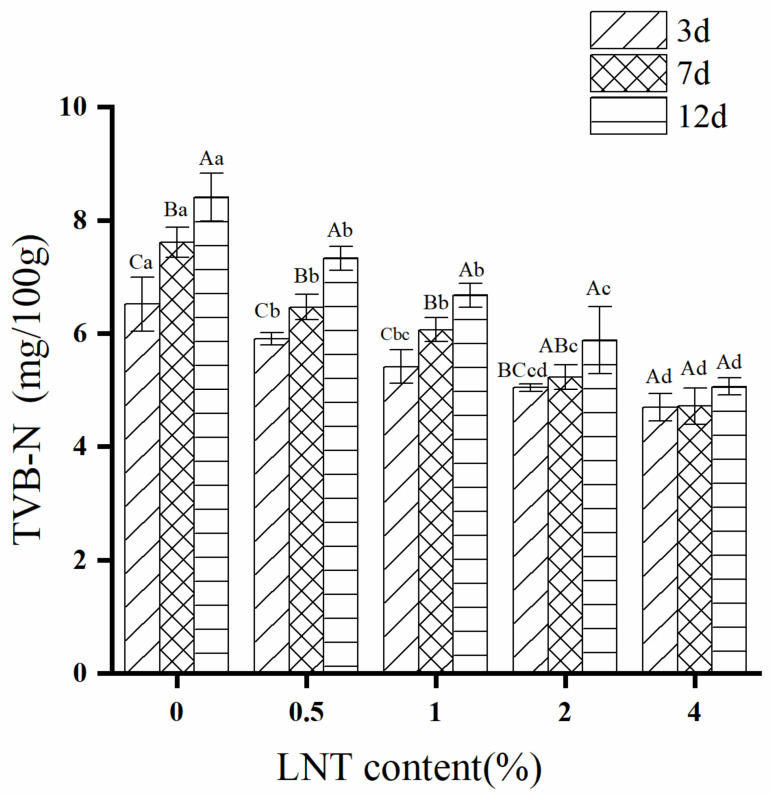
Effects of LNT content and storage time on TVB-N of goose meatballs during cold storage (4 °C). Capital letters indicate significant differences between different storage times for the same sample (*p* ≤ 0.05). Lowercase letters indicate significant differences between different contents of LNT sample during the same storage time (*p* ≤ 0.05).

As one of the most widely used methods to assess the degree of decomposition of meat, TVB-N is the key index of freshness of meat [41]. The two-way ANOVA showed that the addition of LNT significantly affected sample TVN-B and that this effect was significantly influenced by storage time (see the interaction between factors LNT content × storage time in Table S1). The effects of LNT content and storage time on TVB-N of goose meatballs during cold storage (4 °C) are presented in Figure 2. During the whole storage period, the TVB-N of goose meatballs without LNT was significantly increased, which indicated that the samples gradually deteriorated with the prolongation of storage time. This result also explains an overall upward trend in pH at day 7 storage (Table 1). The increase in TVB-N value is related to the activity of endogenous enzymes and spoilage bacteria [42]. However, there was no significant difference in TVB-N at 4% level of LNT during all storage periods. Meanwhile, the TVB-N of the sample decreased (*p* ≤ 0.05) and a dose dependence was showed with the addition of LNT. These results indicate that LNT can effectively inhibit the metabolism of microorganisms and protein decomposition and delay the spoilage of meatballs during storage. LNT as an acidic polysaccharide, may have the effect on inhibiting the microbial decomposition of goose proteins. 

### 3.4. Microstructure and Df

**Figure 3:**
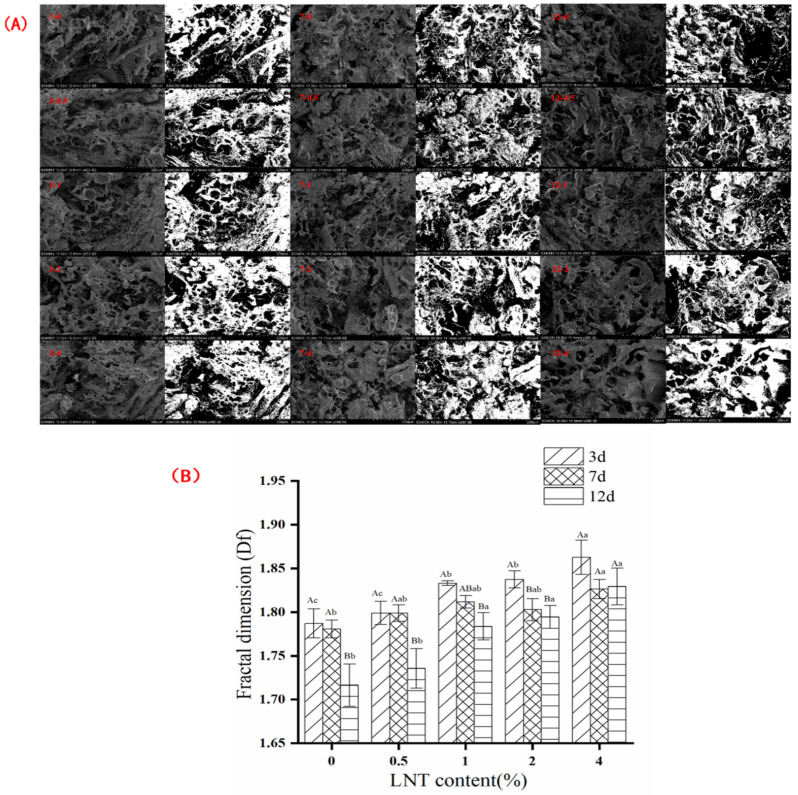
(**A**) Microstructure of goose meatballs and their corresponding binarized images with LNT content and storage time during cold storage (4 °C). The first number 3, 7, 12 represents the storage days, and the second number 0, 0.5, 1, 2, 4 represents the content of LNT. (**B**) Fractal dimension (Df) calculated using the box counting method from SEM binary images of goose meatballs. Capital letters indicate significant differences between different storage times for the same sample (*p* ≤ 0.05). Lowercase letters indicate significant differences between different contents of LNT sample during the same storage time (*p* ≤ 0.05).

Figure 3A shows the microstructural changes in goose meatballs and their corresponding binarized images with different LNT content and storage time during cold storage (4 °C). In this study, the Df of the binary graph was calculated for each sample (Figure 3B), which can objectively and quantitatively compare the differences between the microstructure of goose meatballs [22]. From Figure 3B, the Df values of the LNT-free and 0.5% LNT groups significantly decreased storage of 12 days (Figure 3A (12-0, 12-0.5)) compared with those of 3 days and 7 days storage (Figure 3A (3-0 and 3-0.5, 7-0 and 7-0.5)), indicating the structure of the samples became loose and more pores were appeared after 12 days. This result may be due to the occurrence of oxidation which altered the original structure of samples [43]. With the increase in LNT concentration, the Df of samples showed an increasing trend and the microstructure tends to be more compact during the storage period. Specifically, the Df of the samples in the 4% LNT group increased by 4% compared with the LNT-free sample storage of 3 days (*p* ≤ 0.05). In other words, the 4% LNT group (Figure 3A (3-4)) presented denser structure than the LNT-free samples (Figure 3A (3-0)), which is beneficial to improve the water-retaining ability of goose meatballs [44]. This may because LNT has antioxidant effect which can protect the goose meat protein from denaturation, thus resulting in dense and uniform network structure of goose meatball [45]. In previous studies, the same results were obtained when antioxidants were added to meat to improve the oxidative stability of various meat products [46,47]. In addition, the Df of 4% LNT group did not significantly change during the whole storage period, indicating that excessive LNT can protect the dense and uniform structure of goose meatball during storage. Sensory evaluation results also confirmed the above results, the samples of adding LNT obtained higher scores on appearance attributes, and with the extension of storage time, the scores gradually decreased (Figure S1).

### 3.5. Texture Profile Analysis

TPA is a deformation test to simulate chewing in the human mouth. TPA parameters (hardness, springiness, cohesiveness, and chewiness) offer additional information about the textural characteristics of the meat products [48]. Table 2 presents the effects of LNT content and storage time on texture of goose meatballs during cold storage (4 °C). In general, the hardness, springiness, cohesiveness, and chewiness of goose meatballs showed a decreasing trend during the whole storage period. This result may be attributed to the decomposition of microorganisms and fat oxidation, which resulted in the enhancement of meat tenderness and a change in the texture of goose meatballs [23,49]. The addition of LNT affected the texture profile of goose meatballs. Adding LNT could significantly decrease the hardness of goose meatballs compared with the LNT-free sample. In particular, the hardness of 4% LNT group decreased by 18.49, 20.75 and 28.61% compared with the LNT-free sample during 3-, 7- and 12-days storage, respectively. This outcome is consistent with the report by Wang et al. [50] that the addition of Lentinula edodes in sausage led to a decrease in hardness. In addition, a 2-factor ANOVA showed that there was significant interaction effect of the LNT content × storage time for hardness (*p* = 0.003) of the all sample (Table S1). In this study, the presence of LNT did not affect the springiness and cohesiveness of goose meatballs (*p* ≥ 0.05), but certain concentrations of LNT (0.5%, 1%, and 2%) would increase the chewiness of samples (*p* ≤ 0.05) during the storage of 3 days.

### 3.6. Electronic Tongue Analysis

**Figure 4:**
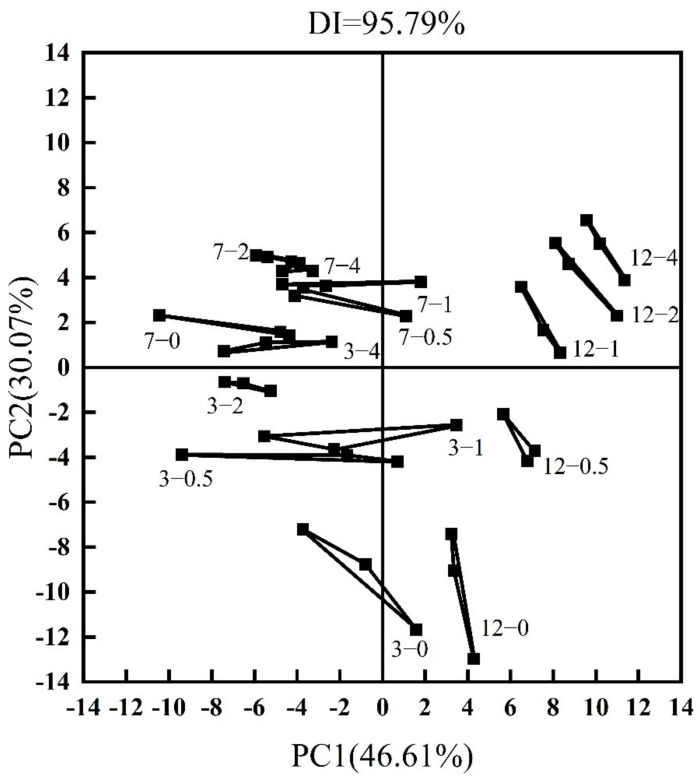
Electronic tongue results of goose meatballs with LNT content and storage time during cold storage (4 °C). The first number 3, 7, 12 represents the storage days, and the second number 0, 0.5, 1, 2, 4 represents the content of LNT.

The principal component score graph is based on a scatter plot, in which each point represents a sample, and the distance between the points represents the magnitude of the feature difference between the samples [24,51]. The PCA of goose meatballs with LNT content and storage time during cold storage (4 °C) is shown in Figure 4. The total contribution rate of PC1 and PC2 is 76.68%, indicating that the 2 components do not represent the overall information of the sample. However, in general, samples stored for 3 days were distributed in the third quadrant (Figure 4. 3-0, 3-0.5, 3-1 and 3-2), samples stored for 7 days were located in the second quadrant stored for 7 days (Figure 4. 7-0, 7-0.5, 7-1, 7-2 and 7-4) and samples stored for 12 days were mostly in the first and the fourth quadrant (Figure 4. 12-0, 12-0.5, 12-1, 12-2 and 12-4), indicating that storage time changed the taste of goose meatballs. In addition, during the storage, LNT concentrations showed a characteristic difference between samples. These results were consistent with the taste attributes of sensory results, storage time and LNT content had different degrees of influence on the taste of all samples (Table S1). 

### 3.7. Changes in Flavor Substances

**Figure 5:**
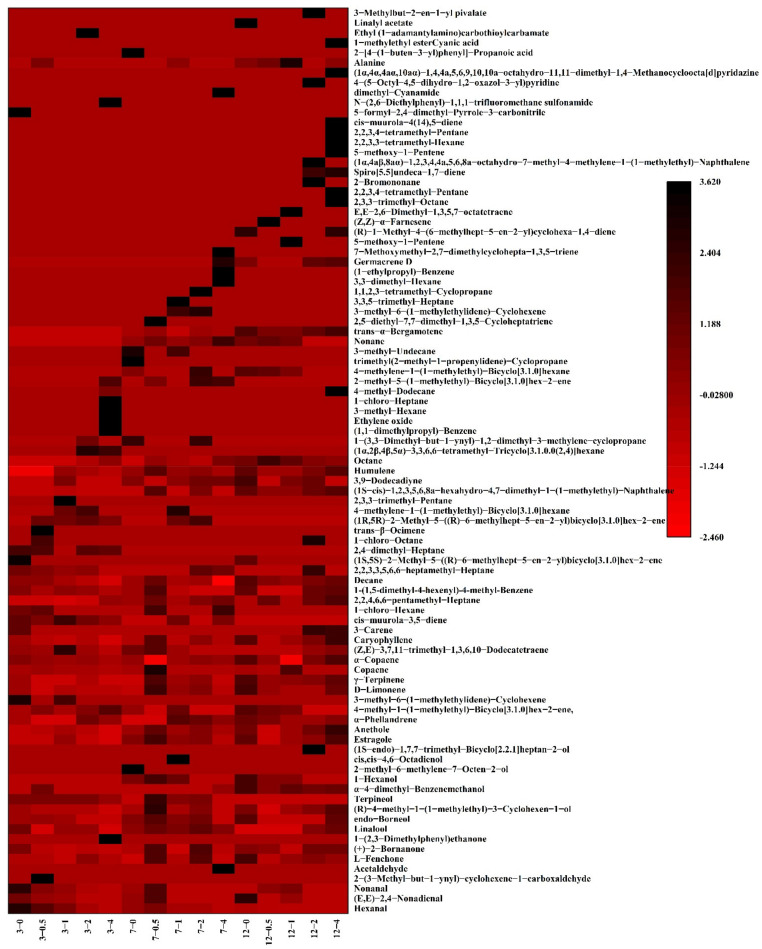
Heatmap display of goose meatballs measured by GC-MS.

A SPME-GC-MS method was used to extract and detect the volatile substances of goose meatballs with different contents of LNT during different storage times. The Heat map was used to show the changes in volatile flavor substance contents in goose meatballs with different LNT concentrations during storage (Figure 5). There were 92 substances preliminarily identified from different groups of goose meatballs, including 5 aldehydes, 61 hydrocarbons, 3 ketones, 9 alcohols, 3 ethers and 11 others. Table 3 statistically analyzes the types and quantities of compounds identified in different groups of goose meatball samples. According to the statistical results, the addition of LNT did not change the total amount of volatile substance in goose meatballs in 3-day storage. However, during 7 and 12 days of storage, the total amount of volatile substances changed in different treatment groups, especially at day 12, there was a difference of 10 substances between the 0.5% LNT and 4% LNT groups. This may because the formation of these volatile compounds was related to the degradation of lipids and proteins through non-enzymatic and enzymatic reactions [52].

Table 4 statistically analyzes the percentage of identified compounds in goose meatball samples with different LNT concentrations during storage. Aldehydes make a great contribution to meat flavor because of its strong volatility and low threshold, but a higher content will bring a negative effect on meat flavor [53]. Among aldehydes, the components with high relative content are hexanal and nonanal. Aldehydes are produced by the oxidation of unsaturated fatty acids, which usually represents the degree of fat oxidation [54]. With the addition of LNT, the content of aldehydes decreased (*p* ≤ 0.05), especially with high concentrations of LNT (4% LNT). There were no aldehydes detected in the 4% LNT samples during storage, indicating that LNT could effectively inhibit fat oxidation, and also had a positive effect on the flavor of goose meatballs.

The threshold of hydrocarbons is high, and individual hydrocarbon has little contribution to meat flavor [55]. However, the synergistic effect of various alkanes and olefins may produce a change in meat flavor. In this study, three kinds of ketones were detected, L-Fenchone, (+)-2-Bornanone and 1-(2,3-Dimethylphenyl) ethenone. There was no change in ketones content in both 0.5% LNT and 1% LNT groups (*p* ≥ 0.05), but an increase in ketones was found in 2% and 4% groups (*p* ≤ 0.05) compared with LNT-free samples after 3 days. However, when the samples were stored for 7 and 12 days, the effect of LNT on ketones content was negligible compared with LNT-free sample. This may because ketones are generally considered to be by-products formed during the degradation of alkanes and the dehydrogenation of secondary alcohols [56]. The relative content of ketones in goose meatballs is low, therefore their contribution to the flavor of goose meatballs is not prominent.

The flavor threshold of alcohol is related to its saturation. Unsaturated alcohol has a lower flavor threshold and influences the meat flavor, while saturated alcohol has a higher flavor threshold and has little effect on the formation of meat flavor [57]. Linalool and terpineol are dominant compounds among the alcohols. With the increase in LNT content, alcohol content showed a decreasing trend during storage. Compared to the control group, the alcohol content in the 4% LNT group decreased from 9.55 to 1.55% and from 12.02 to 1.80% after 7 and 12 days, respectively. This may because the production of alcohols is not only related to microbial fermentation, but also related to fat oxidation [54]. Two ethers were identified in the samples: estragole and anethole. However, these two ethers are the main components of spices, so they have little effect on the flavor of goose meat after cooking.

### 3.8. Correlation Analysis

**Figure 6:**
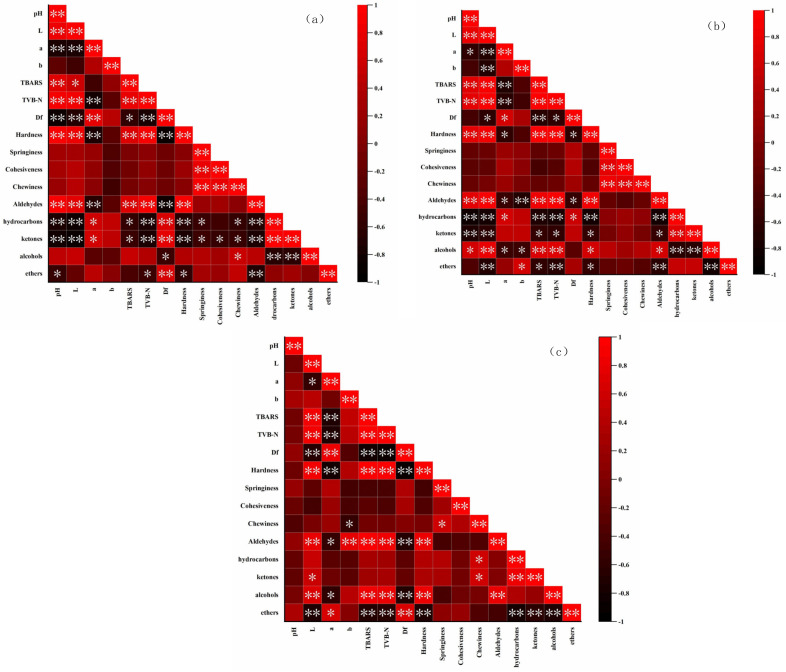
Correlation analysis among characters of adding LNT to goose meatballs. (**a**) represents day 3, (**b**) represents day 7, and (**c**) represents day 12. * represents *p* < 0.05, ** represents *p* < 0.01.

In order to further understand the influence of quality parameters of goose meatballs, correlation analysis was carried out on each index of goose meatballs, and the results are shown in Figure 6. TBARS are often used to assess the degree of fat oxidation in meat products. From Figure 6, the TBARS was positively correlated with L*, TVB-N, hardness and aldehydes, but negatively correlated with Df during the whole storage time. In other words, with the decrease in TBARS, the L*, hardness and aldehyde values of the sample were significantly decreased, while the Df significantly increased, indicating that LNT improved the color, texture, surface structure, flavor and other qualities of the goose meatball samples through inhibiting the fat oxidation during cold storage.

## 4. Conclusions

The lipid oxidation and quality of goose meatballs was affected by LNT content and storage time. Adding LNT can significantly reduce the TBARS in goose meatballs and delay the oxidation of fat during the whole storage period. At the same time, the TVB-N was reduced (*p* ≤ 0.05) and the metabolic activities of microorganisms were inhibited, thus reducing the generation of nitrogenous substances. In addition, the existence of LNT improved the microstructure of goose meatballs and produced characteristic differences in the taste of goose meatballs. Sensory results showed that adding LNT to goose meatballs also improved overall acceptance. These results suggested that LNT can be added to meat products as a new type of natural antioxidant, which can slow down the oxidation process of meat products and, at the same time, improve the quality characteristics of meat products.

## Figures and Tables

**Table 1:** Effects of LNT content and storage time on pH and color changes in goose meatballs during cold storage (4 °C).

	Times/Day	0%	0.5%	1%	2%	4%
pH	3	6.073 ± 0.006 ^Aa^	6.027 ± 0.006 ^Bb^	6.010 ± 0.010 ^Ab^	5.947 ± 0.006 ^Bc^	5.930 ± 0.026 ^Ac^
	7	6.083 ± 0.006 ^Aa^	6.070 ± 0.017 ^Aab^	6.030 ± 0.010 ^Aabc^	6.017 ± 0.031 ^Abc^	5.990 ± 0.052 ^Ac^
	12	5.013 ± 0.035 ^Ba^	4.953 ± 0.011 ^Ca^	4.997 ± 0.063 ^Ba^	5.007 ± 0.015 ^Ca^	5.007 ± 0.038 ^Ba^
L*	3	63.08 ± 0.78 ^Aa^	62.60 ± 1.47 ^Aa^	60.72 ± 2.37 ^Aab^	58.41 ± 0.35 ^Abc^	56.15 ± 2.04 ^Ac^
	7	62.58 ± 0.46 ^Aa^	62.20 ± 0.10 ^Aa^	59.26 ± 0.46 ^Ab^	56.77 ± 1.33 ^Ac^	54.63 ± 0.59 ^Ad^
	12	60.55 ± 0.74 ^Ba^	60.36 ± 3.29 ^Ba^	55.84 ± 1.04 ^Bb^	53.75 ± 1.84 ^Bbc^	51.72 ± 1.13 ^Bc^
A*	3	3.02 ± 0.25 ^Ab^	3.11 ± 0.20 ^Ab^	3.23 ± 0.56 ^Aab^	3.56 ± 0.62 ^Aab^	3.87 ± 0.19 ^Aa^
	7	2.33 ± 0.65 ^Bc^	2.68 ± 0.58 ^Abc^	3.14 ± 0.25 ^Aab^	3.26 ± 0.29 ^Aab^	3.46 ± 0.23 ^Ba^
	12	1.50 ± 0.10 ^Cc^	1.84 ± 0.51 ^Abc^	2.46 ± 1.03 ^Aab^	3.03 ± 0.10 ^Aa^	3.04 ± 0.31 ^Ca^
B*	3	17.96 ± 0.97 ^Aab^	12.36 ± 1.93 ^Bc^	16.93 ± 0.49 ^Bb^	17.54 ± 0.24 ^Bab^	18.78 ± 0.57 ^Ba^
	7	18.48 ± 0.32 ^Ab^	15.31 ± 0.23 ^Ac^	19.20 ± 1.05 ^Aab^	19.57 ± 0.65 ^Aa^	20.11 ± 0.61 ^Aa^
	12	18.95 ± 0.88 ^Aa^	17.82 ± 0.21 ^Ab^	17.82 ± 0.73 ^Bb^	18.09 ± 0.45 ^Bab^	17.99 ± 0.45 ^Bb^
ΔE	3		10.95 ± 1.96 ^c^	12.52 ± 2.72 ^c^	22.18 ± 2.11 ^b^	35.97 ± 4.05 ^a^
	7		4.94 ± 0.73 ^c^	7.86 ± 0.63 ^c^	21.93 ± 2.76 ^b^	31.13 ± 2.41 ^a^
	12		2.45 ± 1.21 ^c^	4.62 ± 0.47 ^c^	16.93 ± 0.99 ^b^	21.76 ± 4.02 ^a^

All values are expressed as mean ± standard deviation. Capital letters indicate significant differences between different storage times for the same sample (*p* ≤ 0.05). Lowercase letters indicate significant differences between different contents of LNT sample during the same storage time (*p* ≤ 0.05).

**Table 2:** Effects of LNT content and storage time on texture of goose meatballs during cold storage (4 °C).

	Times/d	0%	0.5%	1%	2%	4%
Hardness (N)	3	4182.76 ± 88.46 ^Aa^	3969.41 ± 82.55 ^Ab^	3724.42 ± 111.21 ^Ac^	3496.33 ± 149.70 ^Ad^	3409.05 ± 102.60 ^Ad^
	7	4033.74 ± 77.62 ^Aa^	3638.09 ± 11.31 ^Bb^	3547.10 ± 43.76 ^Ab^	3383.15 ± 110.56 ^Ac^	3196.83 ± 127.40 ^Bd^
	12	3861.21 ± 65.21 ^Ba^	3654.51 ± 121.06 ^Bb^	3150.50 ± 111.94 ^Bc^	2938.62 ± 108.77 ^Bd^	2756.58 ± 46.28 ^Ce^
Springiness (mm)	3	0.18 ± 0.02 ^Aab^	0.21 ± 0.04 ^Aab^	0.24 ± 0.06 ^Aa^	0.19 ± 0.02 ^Aab^	0.16 ± 0.01 ^Ab^
	7	0.10 ± 0.01 ^Bb^	0.12 ± 0.01 ^Bb^	0.15 ± 0.00 ^Ba^	0.11 ± 0.01 ^Bb^	0.12 ± 0.01 ^Bb^
	12	0.09 ± 0.01 ^Ba^	0.10 ± 0.01 ^Ba^	0.10 ± 0.00 ^Ba^	0.10 ± 0.01 ^Ba^	0.10 ± 0.01 ^Ca^
Cohesiveness (ratio)	3	0.16 ± 0.00 ^Aab^	0.19 ± 0.03 ^Aa^	0.20 ± 0.02 ^Aa^	0.17 ± 0.03 ^Aab^	0.15 ± 0.00 ^Ab^
	7	0.08 ± 0.01 ^Bb^	0.10 ± 0.02 ^Bb^	0.12 ± 0.00 ^Ba^	0.10 ± 0.00 ^Bb^	0.09 ± 0.01 ^Bb^
	12	0.08 ± 0.01 ^Ba^	0.08 ± 0.01 ^Ba^	0.09 ± 0.01 ^Ba^	0.09 ± 0.00 ^Ba^	0.09 ± 0.00 ^Ba^
Chewiness (N·mm)	3	101.72 ± 3.12 ^Ab^	159.54 ± 17.03 ^Aa^	181.97 ± 28.46 ^Aa^	149.17 ± 27.15 ^Aa^	80.60 ± 12.79 ^Ab^
	7	29.73 ± 4.08 ^Bc^	36.69 ± 1.25 ^Bb^	48.47 ± 3.01 ^Ba^	37.72 ± 5.57 ^Bb^	34.01 ± 3.11 ^Bbc^
	12	20.67 ± 6.17 ^Bc^	33.97 ± 0.82 ^Ba^	34.58 ± 1.02 ^Ba^	27.16 ± 2.44 ^Bb^	25.21 ± 3.48 ^Bbc^

All values are expressed as mean ± standard deviation. Capital letters indicate significant differences between different storage times for the same sample (*p* ≤ 0.05). Lowercase letters indicate significant differences between different contents of LNT sample during the same storage time (*p* ≤ 0.05).

**Table 3:** Effects of LNT content and storage time on substance types of goose meatballs during cold storage (4 °C).

	Times/d	0%	0.5%	1%	2%	4%
aldehydes	3	3	4	3	2	0
	7	3	3	1	1	0
	12	2	2	2	0	0
hydrocarbons	3	18	17	19	20	21
	7	18	16	20	20	21
	12	20	15	17	21	24
ketones	3	2	2	2	2	3
	7	2	2	2	2	2
	12	2	2	2	2	2
alcohols	3	4	4	4	3	2
	7	5	5	6	4	3
	12	4	3	3	4	4
ethers	3	2	2	2	2	2
	7	2	2	2	2	2
	12	2	2	2	2	2
others	3	1	1	0	1	1
	7	1	0	1	0	1
	12	2	1	1	2	3
total	3	30	30	30	30	29
	7	31	28	32	29	30
	12	32	25	27	31	35

**Table 4:** Effects of LNT content on substance content of goose meatballs during cold storage (4 °C).

	Times/d	0%	0.5%	1%	2%	4%
Aldehydes/%	3	35.33 ± 1.39 ^a^	25.40 ± 0.74 ^b^	10.04 ± 0.23 ^c^	3.89 ± 0.90 ^cd^	0.00 ± 0.00 ^d^
	7	10.26 ± 0.76 ^a^	9.54 ± 1.90 ^a^	4.43 ± 1.82 ^b^	3.10 ± 0.64 ^b^	0.00 ± 0.00 ^c^
	12	2.32 ± 0.12 ^a^	0.73 ± 0.33 ^b^	0.63 ± 0.17 ^b^	0.00 ± 0.00 ^c^	0.00 ± 0.00 ^c^
hydrocarbons/%	3	27.34 ± 3.81 ^c^	27.60 ± 0.59 ^c^	29.24 ± 2.00 ^bc^	35.70 ± 3.41 ^b^	49.78 ± 2.83 ^a^
	7	24.17 ± 0.12 ^c^	25.72 ± 0.11 ^bc^	28.68 ± 0.66 ^bc^	30.21 ± 3.00 ^b^	36.32 ± 1.64 ^a^
	12	28.45 ± 2.71 ^b^	44.30 ± 4.91 ^a^	33.07 ± 3.14 ^b^	28.26 ± 1.31 ^b^	26.60 ± 1.03 ^b^
ketones/%	3	0.67 ± 0.03 ^c^	0.68 ± 0.00 ^c^	0.69 ± 0.04 ^c^	0.91 ± 0.04 ^b^	1.14 ± 0.05 ^a^
	7	0.55 ± 0.02 ^bc^	0.53 ± 0.04 ^c^	0.59 ± 0.09 ^bc^	0.79 ± 0.05 ^a^	0.72 ± 0.04 ^ab^
	12	0.59 ± 0.05 ^b^	0.89 ± 0.19 ^a^	0.73 ± 0.09 ^ab^	0.71 ± 0.02 ^ab^	0.46 ± 0.03 ^b^
alcohols/%	3	1.85 ± 0.31 ^a^	1.90 ± 0.01 ^a^	1.80 ± 0.22 ^ab^	1.81 ± 0.41 ^ab^	1.20 ± 0.18 ^b^
	7	9.55 ± 2.23 ^a^	10.88 ± 1.44 ^a^	12.11 ± 1.88 ^a^	2.25 ± 0.10 ^b^	1.55 ± 0.16 ^b^
	12	12.02 ± 3.02 ^a^	10.36 ± 3.59 ^a^	6.79 ± 0.98 ^ab^	1.81 ± 0.27 ^b^	1.80 ± 0.06 ^b^
ethers/%	3	34.82 ± 3.52 ^d^	44.01 ± 0.79 ^c^	58.27 ± 3.78 ^a^	56.44 ± 4.11 ^ab^	47.60 ± 2.84 ^bc^
	7	55.24 ± 1.24 ^b^	54.67 ± 4.00 ^b^	54.04 ± 0.40 ^b^	61.33 ± 1.61 ^a^	61.05 ± 1.91 ^a^
	12	55.79 ± 1.11 ^b^	43.18 ± 3.91 ^c^	57.71 ± 1.15 ^b^	69.14 ± 1.88 ^a^	70.94 ± 1.95 ^a^
others/%	3	0.00 ± 0.00 ^c^	0.42 ± 0.09 ^b^	0.00 ± 0.00 ^c^	1.24 ± 0.15 ^a^	0.27 ± 0.24 ^bc^
	7	0.24 ± 0.04 ^a^	0.00 ± 0.00 ^b^	0.16 ± 0.02 ^ab^	0.00 ± 0.00 ^b^	0.09 ± 0.01 ^ab^
	12	0.84 ± 0.03 ^ab^	0.54 ± 0.13 ^ab^	1.08 ± 0.02 ^a^	0.09 ± 0.01 ^b^	0.20 ± 0.02 ^ab^

All values are expressed as mean ± standard deviation. Lowercase letters indicate significant differences between different contents of LNT sample in the same storage time (*p* ≤ 0.05).

## Data Availability

The data presented in this study is available on request from the corresponding author.

## Supplementary Materials

The following supporting information can be downloaded at: https://www.mdpi.com/article/10.3390/foods11071055/s1, Figure S1: Effect of LNT on the sensory attributes of the goose meatball; Table S1: *p* value of two-way ANOVA.
