# Supplementary material for: Effect of Lentinan on Lipid Oxidation and Quality Change in Goose Meatballs during Cold Storage
Source: Foods. 2022 Apr 6;11(7):1055. doi: 10.3390/foods11071055 (PMC8997726; doi:10.3390/foods11071055)
Supplement: Supplementary file 1 [file foods-11-01055-s001.zip › foods-1656889-supplementary.pdf]

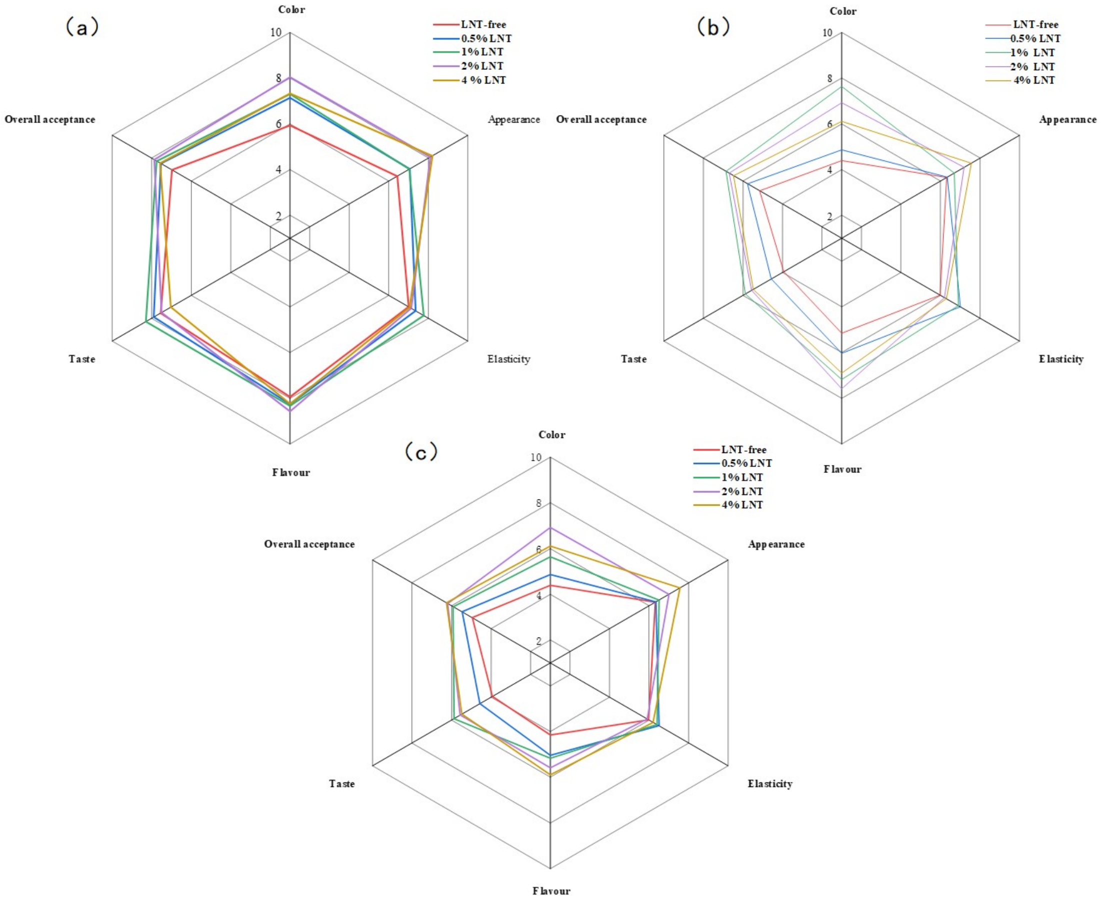

Figure S1. Effect of LNT on the sensory attributes of the goose meatball.

Table S1. P value of two-way ANOVA.

	**LNT content**	**Storge time**	**LNT content×Storge time**
pH	< 0.001	< 0.001	0.001
L*	< 0.001	< 0.001	0.651
a*	< 0.001	< 0.001	0.533
b*	< 0.001	< 0.001	< 0.001
TBARS	< 0.001	< 0.001	< 0.001
TVB-N	< 0.001	< 0.001	0.001
Df	< 0.001	< 0.001	0.117

Hardness(N)	< 0.001	< 0.001	0.003
Springiness(mm)	0.001	< 0.001	0.146
Cohesiveness(ratio)	0.001	< 0.001	0.243
Chewiness(N*mm)	< 0.001	< 0.001	< 0.001
